# Regional cerebral oxygen saturation variability and brain injury in preterm infants

**DOI:** 10.3389/fped.2024.1426874

**Published:** 2024-07-22

**Authors:** Tomislav Ćaleta, Martin J. Ryll, Katarina Bojanić, Nada Sindičić Dessardo, Darrell R. Schroeder, Juraj Sprung, Toby N. Weingarten, Milan Radoš, Ivica Kostović, Ruža Grizelj

**Affiliations:** ^1^ Department of Pediatrics, School of Medicine University of Zagreb, University Hospital Centre Zagreb, Zagreb, Croatia; ^2^ Department of Anesthesiology and Perioperative Medicine, Mayo Clinic College of Medicine and Science, Rochester, MN, United States; ^3^ Division of Neonatology, Department of Obstetrics and Gynecology, University Hospital Merkur, Zagreb, Croatia; ^4^ Health Sciences Research, Division of Epidemiology, Mayo Clinic College of Medicine and Science, Rochester, MN, United States; ^5^ Croatian Institute for Brain Research, School of Medicine University of Zagreb, Zagreb, Croatia; ^6^ Center for Research on Perinatal Etiopathogenesis of Neurological and Cognitive Diseases, School of Medicine University of Zagreb, Zagreb, Croatia

**Keywords:** neonates, preterm infants, magnetic resonance imaging, intraventricular hemorrhage, white matter injury, near-infrared spectroscopy, regional cerebral oxygen saturation

## Abstract

**Objective:**

To examine whether variation of regional cerebral oxygen saturation (rScO_2_) within three days after delivery predicts development of brain injury (intraventricular/cerebellar hemorrhage or white matter injury) in preterm infants.

**Study design:**

A prospective study of neonates <32 weeks gestational age with normal cranial ultrasound admitted between 2018 and 2022. All received rScO_2_ monitoring with near-infrared spectroscopy at admission up to 72 h of life. To assess brain injury a magnetic resonance imaging was performed at term-equivalent age. We assessed the association between rScO_2_ variability (short-term average real variability, rScO_2ARV_, and standard deviation, rScO_2SD_), mean rScO_2_ (rScO_2MEAN_), and percentage of time rScO_2_ spent below 60% (rScO_2TIME<60%_) during the first 72 h of life and brain injury.

**Results:**

The median [IQR] time from birth to brain imaging was 68 [59-79] days. Of 81 neonates, 49 had some form of brain injury. Compared to neonates without injury, in those with injury rScO_2ARV_ was higher during the first 24 h (*P *= 0.026); rScO_2SD_ was higher at 24 and 72 h (*P *= 0.029 and *P *= 0.030, respectively), rScO_2MEAN_ was lower at 48 h (*P *= 0.042), and rScO_2TIME<60%_ was longer at 24, 48, and 72 h (*P *= 0.050, *P *= 0.041, and *P *= 0.009, respectively). Similar results were observed in multivariable logistic regression. Although not all results were statistically significant, increased rScO_2_ variability (rScO_2ARV_ and rScO_2SD_) and lower mean values of rScO2 were associated with increased likelihood of brain injury.

**Conclusions:**

In preterm infants increased aberration of rScO_2_ in early postdelivery period was associated with an increased likelihood of brain injury diagnosis at term-equivalent age.

## 1 Introduction

Brain injury in the preterm infant results from the combined developmental and destructive effects on the maturing nervous system due to multisystemic diseases and conditions from prenatal to postnatal life (1). Pre-conceptional maternal toxic stress and pregnancy-related illnesses affecting the maternal-placental-fetal triad can disrupt fetal brain development, contributing to preterm birth and/or increasing risks for peripartum brain injuries (1, 2). Various postnatal injurious triggers such as respiratory insufficiency and hemodynamic instability secondary to severe respiratory disease, recurrent apneic spells, hemodynamically significant ductus arteriosus, late-onset sepsis or conditions such as necrotizing enterocolitis further increase the risk and promote subsequent brain injury (3, 4).

Intracranial hemorrhage and white matter injury (WMI) are frequent pathologies (20%–30%) in preterm infants (5–8). Intraventricular hemorrhage (IVH) usually originates in the subependymal germinal matrix, a richly vascularized collection of neuronal-glial precursor cells in the developing brain (9). The risk of hemorrhage is inversely proportional to gestational age (GA), with most of IVH occurring in infants less than 32 weeks of gestation (10). Factors primarily related to dysregulation of cerebral blood flow and pressure in the microvascular bed of the germinal matrix play a major contributory pathogenic role (9, 11, 12). Most IVH events occur within the first week of delivery, and in the majority (90%) can be detected within the first 72 h of life (13). Cerebellar hemorrhage is also a common form of brain injury in preterm infants. Detection of these injuries by magnetic resonance imaging (MRI) has been reported in up to 37% of infants less than 33 weeks GA (14). WMI represents a spectrum of disease that ranges from focal necrotic lesions deep in the white matter, with or without subsequent cyst formation, to the more common, diffuse, and nondestructive WMI (15). The injury is believed to be induced by cerebral ischemia, infection and/or inflammation (16). Several fundamental physiological factors related to cerebral blood flow, including oxygenation, hypocarbia, levels of glucose and its metabolites, and a variety of inflammatory factors, likely influence the severity of WMI (17).

Considering that prevalent types of brain injury among preterm infants often coincide with hypoxic, ischemic, and reperfusion events in the early postdelivery period, it is of utmost importance to be able to assess adequacy of cerebral blood flow to improve managements designed to mitigate the risk for injury. Near-infrared spectroscopy (*N*IRS) monitors regional cerebral oxygen saturation (rScO_2_), provides non-invasive information on hemodynamics, real time brain oxygen delivery (18), and is considered to be a surrogate marker for cerebral blood flow (19–21). NIRS uses multiple wavelengths of near-infrared light and relies on the absorption spectra of oxygenated and deoxygenated hemoglobin to calculate relative concentrations of each, which are then used to calculate rScO_2_. Since NIRS makes no distinction between brain blood compartments, rScO_2_ estimates hemoglobin oxygen saturation in a mixed arterial, capillary, and venous compartments (22). The association between rScO_2_ measurements and development of brain injury is not well explored. In the current study we hypothesize that variability in rScO_2_ recorded from NIRS in the early postdelivery period may predict brain injury assessed from MRI at term-equivalent age (TEA). We especially focus on short-term average real variability (ARV) of rScO_2_ during early postdelivery period as a potential culprit for brain injury. This hypothesis was tested on preterm infants by rScO_2_ monitoring with NIRS for the first 72 h after birth. An improved understanding of the relationship between altered rScO_2_ and development of brain injury may be used in future management strategies designed to improve neonatal outcomes.

## 2 Methods

### 2.1 Settings

This study was conducted in the University Hospital Centre (UHC) and the Croatian Institute for Brain Research, Zagreb, Croatia as a part of a multidisciplinary, longitudinal research project. The UHC is the largest Croatian tertiary referral center for neonatal care and does not have a maternity ward. The hospital admits preterm infants from hospitals that do have maternity wards but do not have the capacity to manage high-risk neonates. Therefore, all neonates in the current study are outborns.

### 2.2 Patient population, inclusion/exclusion criteria

This is a prospective study of all consecutive newborn admissions to the Neonatal Intensive Care Unit (NICU) at the UHC Zagreb between May 1st, 2018, and June 31st, 2022. The infants were eligible for enrollment if they were less than 32 weeks’ GA at birth and had a normal cranial ultrasound (cUS) on admission. Preterm infants with chromosomal or congenital anomalies, and those with delayed transfer (>12 h) from outside institutions were excluded.

### 2.3 Study design, NIRS monitoring and study aims

Upon NICU admission cUS was performed to exclude the presence of brain injury. All qualified infants (i.e., no brain injury on cUS) received rScO_2_ monitoring using NIRS immediately on admission for up to 72 h of life. A 72-hour period has been accepted as a suitable time frame for NIRS monitoring in premature infants (23), as majority of IVH in premature infants happens within the first 3 days of life (24). In our study a two wavelength (730 and 810 nm) near-infrared spectrometer (INVOS 5100, Covidien, Mansfeld, MA) was used by firmly attaching a small neonatal sensor (Covidien, Mansfield, MA) on the left side of the infant's forehead. Four rScO_2_ summary statistics were considered: (1) short-term rScO_2_ average real variability (rScO_2ARV_) using following equation:

$${\mathrm{rScO}}_{2\mathrm{AVR}}=\frac{1}{N-1}\overset{N-1}{\underset{k=1}{\sum }}|{\mathrm{rScO}}_{2k+1}-{\mathrm{rScO}}_{2k}|$$

(2) rScO_2_ standard deviation (rScO_2SD_), (3) rScO_2_ mean (rScO_2MEAN_), and 4) the percentage of time neonate spent with rScO_2_<60% (rScO_2TIME<60%_) all during 72 h after birth. The primary aim was to assess the association between variability of rScO_2_ (rScO_2ARV_ and rScO_2SD_) and brain injury, and secondary aims were to assess the association between average rScO_2_, (rScO_2MEAN_) and percentage of time spent at oxygen saturation below 60% (rScO_2TIME<60%_) and brain injury.

### 2.4 Data collection

We reviewed obstetric, demographic and neonatal data from the hospital records: sex, GA, birth weight, type of delivery (natural delivery vs. Cesarean section), antenatal corticosteroid treatment, Apgar scores, age at hospital admission, Scores for Neonatal Acute Physiology Perinatal Extension II (SNAPPE-II); variables related to treatment: primary respiratory support, duration of mechanical ventilation, surfactant administration, transfusion of blood and blood products, use of inotropes; and prematurity related complications: pneumothorax, bronchopulmonary dysplasia, necrotizing enterocolitis, infection/sepsis, retinopathy of prematurity.

### 2.5 Grading of brain injuries

Brain imaging at TEA was done using a 3T MRI scanner (Magnetom, Prisma^FIT^, Siemens). MRI scanning was performed after regular feeding, infants were wrapped with linen diapers and a blanket. A neuroradiologist blinded to clinical data evaluated the MRI findings. WMI grades considered are: Grade I—punctate lesions; Grade II—small periventricular cysts; Grade III—extensive periventricular cysts; Grade IV—extensive subcortical cysts—also called multicystic encephalomalacia (25–27). IVH was classified according to Papile classification: Grade I—hemorrhage limited to germinal matrix; Grade II—extension into normal-sized ventricles; Grade III—extensive hemorrhage with dilatation of the ventricles; Grade IV—parenchymal involvement (28, 29). Cerebellar hemorrhage was noted as present or absent.

### 2.6 Statistical analysis

Raw rScO_2_ NIRS measurements were recorded every 5–15 s. To exclude outliers, we aggregated raw rScO_2_ data as a mean over 5-minute intervals for the four features of interest. The aggregated rScO_2_ measurements were analyzed for 24-, 48-, and 72-hour intervals following birth. We examined the association between four rScO_2_ features (time-weighted rScO_2ARV_, rScO_2SD_, rScO_2MEAN_, and rScO_2TIME<60%_) and brain injury at TEA. The rScO_2ARV_ was calculated as the average of absolute differences between consecutive rScO_2_ measurements during the observed time frame using a previously described equation (30, 31). rScO_2ARV_ feature accounts for the order in which the respective rScO_2_ measurements occurred and corrects for limitations of the commonly used measures of variability such as standard deviation, which accounts only for the dispersion of values around the mean, and not for the order of the respective readings (31). Patients with >50% missing rScO_2_ values during the predetermined time intervals following admission were assigned a missing value for the respective feature. For calculating the rScO_2ARV_ and rScO_2SD_, the rScO_2_ ceiling-value of 95% was handled by excluding any of the aggregated 95% measurements, that were flanked on both sides by 95% measurements. For calculating the rScO_2MEAN_ and the rScO_2TIME<60%_ these 95%-measurements were not excluded.

For univariable analysis, rScO_2_ features were compared between those with and without brain injury using the two-sample *t*-test or Mann Whitney *U*-test as appropriate. For the multivariable logistic regression analysis, our cohort size allows for two covariates aside from our feature of interest, for which we chose GA and birth weight. Results from the multivariable logistic regression model are summarized as odds ratio and 95% confidence interval for the given rScO_2_ feature. There was no evidence of significant non-linearity of GA, birth weight, and all rScO_2_ features, as tested by comparing a univariable linear and a univariable restricted cubic spline model (with knots at the 5th, 50th, and 95th or 15th, 50th, and 95th percentile) via the likelihood ratio test for each variable. A *P*-value <0.05 was determined statistically significant throughout. All statistical analyses were performed with Python v.3.9 (Python Software Foundation, Wilmington, Delaware, USA).

## 3 Results

### 3.1 Cohort characteristics

**Figure 1:**
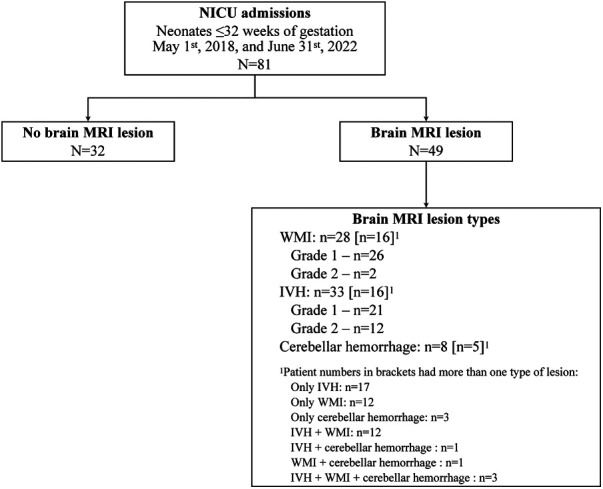
Summary of 81 preterm infants according to MRI results at term-equivalent age. IVH, intraventricular hemorrhage; WMI, white matter injury.

Between May 1st, 2018, and June 31st, 2022, 81 neonates met criteria and were included in the study. The median [IQR] time from birth to initiation of NIRS monitoring was 2.5 [1.4–3.6] hours, and from birth to MRI at TEA 68 [59–79] days. MRI at TEA ruled out the presence of brain injury in 32 neonates, while 49 were diagnosed with single or multiple brain injuries (all were mild, grades I or II): 33 (40.7%) neonates had IVH, 28 (34.6%) had WMI, and 8 (10%) had cerebellar hemorrhage (Figure 1). Table 1 shows neonatal characteristics, overall and according to the presence or absence of brain injury. There were no significant differences in characteristics between infants with and without injury: GA at delivery (*P *= 0.555), Apgar scores at 1 and 5 min (*P *= 0.673 and 0.899, respectively), and main comorbidities (sepsis, *P *> 0.99; need for respiratory support, *P *= 0.155; necrotizing enterocolitis, *P *= 0.462; bronchopulmonary dysplasia, *P *= 0.590; or retinopathy, *P *= 0.511).

**Table 1:** Demographic and clinical characteristics of preterm infants in our cohort.

Characteristics	All patients (*N* = 81)	Normal MRI (*n* = 32)	Abnormal MRI (*n* = 49)	*P* value
Sex (male)	39 (48.1%)	17 (53.1%)	22 (44.9%)	0.619
GA at delivery (weeks)	30.1 [28.6–31.6]	30.9 [29.1–31.6]	30.0 [28.0–31.6]	0.555
Delivery (C-section)	44 (54.3%)	18 (56.2%)	26 (53.1%)	0.957
Birth weight (grams)	1,345 [1,125–1,550]	1,315 [1,129–1,513]	1,355 [1,105–1,560]	0.768
Head circumference (cm)	28.0 [26.5–29.0]	28.0 [26.5–29.0]	27.5 [26.0–29.0]	0.454
Apgar score at 1 min	7.0 [3.0–8.0]	7.0 [3.0–8.0]	7.0 [4.0–8.0]	0.673
Apgar score at 5 min	8.0 [6.0–9.0]	7.5 [5.2–9.0]	8.0 [6.0–9.0]	0.899
SNAPPE II score	23.0 [9.0–38.0]	20.5 [5.0–38.8]	23.0 [13.0–34.0]	0.670
Full steroid course received	17 (21.0%)	6 (18.8%)	11 (22.4%)	0.904
Surfactant administered	43 (53.1%)	15 (46.9%)	28 (57.1%)	0.498
Inotropes during first 3 days	15 (18.5%)	4 (12.5%)	11 (22.4%)	0.382
RBC transfusion	11 (13.6%)	3 (9.4%)	8 (16.3%)	0.513
Sepsis	12 (14.8%)	5 (15.6%)	7 (14.3%)	> 0.99
Pneumothorax	8 (9.9%)	3 (9.4%)	5 (10.2%)	> 0.99
Primary respiratory support				0.155
Invasive ventilation	39 (48.1%)	12 (37.5%)	27 (55.1%)	
nCPAP	40 (49.4%)	19 (59.4%)	21 (42.9%)	
None	2 (2.5%)	1 (3.1%)	1 (2.1%)	
Necrotizing enterocolitis				0.462
No	77 (95.1%)	31 (96.9%)	46 (93.9%)	
1st grade	1 (1.2%)	0 (0%)	1 (2.0%)	
2nd grade	2 (2.5%)	0 (0%)	2 (4.1%)	
3rd grade	1 (1.2%)	1 (3.1%)	0 (0%)	
Bronchopulmonary dysplasia				0.590
No	57 (70.4%)	24 (75.0%)	33 (67.3%)	
Mild	22 (27.2%)	8 (25.0%)	14 (28.6%)	
Moderate	2 (2.5%)	0 (0%)	2 (4.1%)	
Retinopathy of prematurity				0.511
No	55 (67.9%)	25 (78.1%)	30 (61.2%)	
1st stage	5 (6.2%)	1 (3.1%)	4 (8.2%)	
2nd stage	17 (21.0%)	5 (15.6%)	12 (24.5%)	
3rd stage	4 (4.9%)	1 (3.1%)	3 (6.1%)	

Continuous variables are presented as median [IQR] and were compared by independent *t*-test or Mann-Whitney *U*-test (depending on whether they were normally distributed). Binary variables are presented as count (percentage) and were compared via Chi-Square or Fisher's Exact test, as appropriate. GA, gestational age; SNAPPE II, Score for Neonatal Acute Physiology with Perinatal Extension-II; nCPAP, nasal continuous positive airway pressure; RBC, red blood cells.

### 3.2 Association of rScO_2_ features with brain injury at TEA

**Figure 2:**
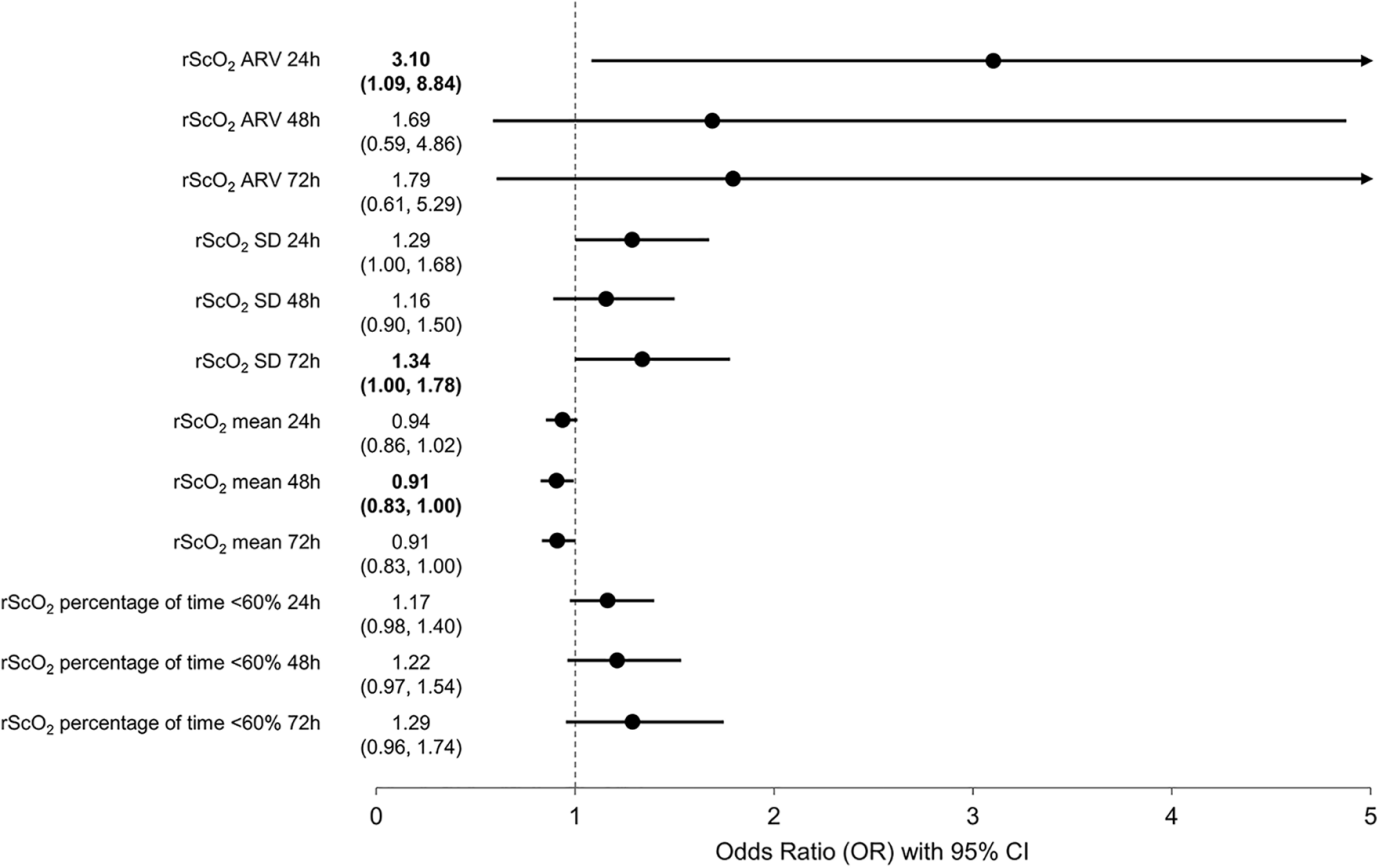
Results of logistic regression analysis showing the association between rScO_2_ features (average real variability; rScO_2ARV_; standard deviation, rScO_2SD_; mean, rScO_2MEAN_; and percentage of time below 60%, rScO_2TIME<60%_) and brain injury at term-equivalent age. Values >1 indicate increased likelihood for brain injury at term-equivalent age.

Of the 81 neonates, 72, 77, and 80 had sufficient rScO_2_ data (continuous NIRS monitoring for >50% of the timeframe) for the 24-, 48-, and 72-hours after birth, respectively. Compared to neonates without brain injury, rScO_2ARV_ was higher during the first 24 h in those diagnosed with brain injury, *P *= 0.026 (Table 2, Figure 2). Similarly, rScO_2SD_ was higher at 24 and 72 h (*P *= 0.029 and *P *= 0.030, respectively) in those with injury. The rScO_2MEAN_ was lower at 48 h (*P *= 0.042) in those with injury, and the percentage of time neonates spent at rScO_2TIME<60%_ was higher in those with injury (*P *= 0.050, *P *= 0.041, and *P *= 0.009 at 24-, 48-, and 72-hours, respectively) (Table 2, Supplementary Figure S1). Similar results were observed from logistic regression analysis adjusted for GA and birth weight (Table 2). Although not all results were significant, increased rScO_2_ variability (rScO_2ARV,_ rScO_2SD_) was consistently associated with increased likelihood of brain injury at TEA. Also, lower values of rScO_2_ (rScO_2MEAN_ and rScO_2TIME<60%_) were associated with increased likelihood of brain injury (Table 2 and Figure 2).

**Table 2:** Comparison of four rScO_2_ features during the first 72 h of life in infants with and without brain injury as seen on magnetic resonance imaging at term-equivalent age.

rScO_2_ features	Hours^a^	All infants^b^ (*N* = 81)	Normal MRI^b^ (*n* = 32)	Abnormal MRI^b^ (*n* = 49)	*P* ^c^	ORs [95% CI]^d^	*P* ^d^
rScO_2ARV_	24	1.9 [1.7–2.3]	1.8 [1.6–2.0]	2.1 [1.7–2.6]	**0**.**026**	**3.1 [1.09–8.84]**	**0**.**034**
	48	2.0 [1.7–2.3]	2.0 [1.6–2.2]	2.0 [1.7–2.3]	0.411	1.69 [0.59–4.86]	0.328
	72	1.9 [1.7–2.3]	1.9 [1.7–2.2]	1.9 [1.8–2.3]	0.364	1.79 [0.61–5.29]	0.289
rScO_2SD_	24	5.7 [4.4–6.8]	5.3 [3.9–6.5]	6.2 [4.5–7.9]	**0**.**029**	1.29 [1.0–1.68]	0.054
	48	6.1 [4.9–7.5]	6.1 [4.6–7.0]	6.1 [5.0–7.7]	0.201	1.16 [0.9–1.5]	0.252
	72	6.5 [5.4–7.7]	6.2 [5.3–6.9]	6.6 [5.4–8.0]	**0**.**030**	**1.34 [1.0–1.78]**	**0**.**046**
rScO_2MEAN_	24	76.6 [73.1–80.0]	77.0 [73.5–82.6]	76.1 [72.2–77.8]	0.132	0.94 [0.86–1.02]	0.120
	48	78.9 [75.4–82.5]	79.9 [76.1–86.4]	78.6 [75.3–80.6]	**0**.**042**	**0.91 [0.83–1.0]**	**0**.**044**
	72	79.4 [77.1–83.1]	80.0 [77.4–86.8]	79.0 [76.4–82.0]	0.054	0.91 [0.83–1.0]	0.054
rScO_2TIME<60%_	24	0.5 [0.0–3.0]	0.0 [0.0–1.8]	0.8 [0.0–4.2]	**0**.**049**	1.17 [0.98–1.4]	0.088
	48	0.2 [0.0–1.8]	0.0 [0.0–0.7]	0.4 [0.0–2.4]	**0**.**041**	1.22 [0.97–1.54]	0.092
	72	0.3 [0.0–1.3]	0.0 [0.0–0.6]	0.5 [0.1–2.5]	**0**.**009**	1.29 [0.96–1.74]	0.090

All values for ARV, SD and mean are per 1% change in rScO_2_; time <60% are per 1% of that timeframe below rScO_2_ of 60%. rScO_2_, Regional cerebral oxygen saturation; rScO_2ARV_, rScO_2_ average real variability; rScO_2SD_, rScO_2_ standard deviation; rScO_2MEAN_, rScO_2_ mean; rScO_2TIME<60%_, percentage of time rScO_2_ was below 60%.

^a^ The duration (hours) of monitoring after birth.

^b^ Data presented as “median [25th percentile—75th percentile]”.

^c^ Univariable comparison via independent *t*-test or Mann-Whitney *U*-test as appropriate.

^d^ Logistic regression model adjusted for both gestational age at delivery and birth weight. Significant values are bolded.

## 4 Discussion

The most important findings of this study are that increased variability of regional cerebral oxygen saturation, lower mean saturation, and longer time neonates spent at saturation below 60% in the early postdelivery period were associated with an increased likelihood for being diagnosed with brain injury at TEA. Our results cannot distinguish if this association is consequential (rScO_2_ pattern reflects presence of injury) or causative (rScO_2_ pattern contributed to development of injury), therefore our study provides direction for future research.

Variations in cerebral perfusion and oxygenation are considered to be the key risk factors for brain injury in preterm infants (9, 11, 12). Continuous assessment of rScO_2_ can identify infants with altered cerebral oxygenation (32). Although NIRS is increasingly used by neonatologists for rScO_2_ monitoring, there are no reports that consistently establish the rScO_2_ references or cut-off values for adverse outcomes related to altered brain oxygenation (23, 33). Alderliesten et al. (23) published reference values of rScO_2_ during the first three days of life in 999 preterm infants (GA <32 weeks) and found that at NICU admission the average rScO_2_ was ∼65% and continued to increase with GA at a mean rate of 1% per week, following a parabolic curve in relation to postnatal age with a peak at −36 h. It is important to note, Alderliesten et al. (23) references were obtained mostly from measurements using small adult sensors (983 small adult sensors and 16 neonatal sensors). In order to convert neonatal sensor readings to the small adult sensor equivalent, the obtained rScO_2_ values were interpolated using statistical modelling tools (23). It is well established that rScO_2_ values depend on the type of NIRS sensor used (e.g., adult, neonatal, pediatric) (23, 34). In comparison to adult sensors, neonatal rScO_2_ sensor readings are consistently higher, but the difference is not fixed and is less at the threshold indicative of cerebral hypoxia; the neonatal sensor difference is approximately 10% when adult sensors read 85%, but nearly similar (58.8%) when adult sensors read 55% (35). The SafeBoosC-III study evaluating the benefit of an interventional strategy to reduce cerebral hypoxia using NIRS-derived rScO_2_ monitoring demonstrate no significant difference between group in rates of death or severe brain injury at 36 weeks post-menstrual age (36). The study used at least 5 different device and sensor combinations with varying hypoxia thresholds based on linear transformations obtained in an *in vitro* model. Although the trial did not show evidence of decreased mortality or severe brain injury, concerns remain that the selected thresholds were not equivalent across devices due to proprietary algorithms and the nonlinear nature of human physiology (37). Therefore, in order to properly interpret rScO_2_ cut-off values, it is of utmost importance to specify the type of sensor when comparing the data between studies.

Because low brain blood flow is associated with reduced oxygenation it poses a risk for development of brain injury. Specifically, Alderliesten et al. (34) found that a rScO_2_<55% (using a small adult sensor) increased risk for grade III/IV IVH with an odds ratio of 1.017 per one percent (95%CI 1.007–1.026, GA corrected) of time spent below 55%, as well as in neonates who spent at least 20% of time below 55% in the first 72 h following delivery. Furthermore, Alderliesten et al. (34) found that a rScO_2_<55% was associated with unfavorable cognitive outcomes at 24 months with an OR of 1.4 (CI 1.1–1.7) for neonates who spent at least 20% of time below that threshold during the first 3 days after delivery. Chock et al. (38) measured rScO_2_ with neonatal sensors and reported that infants with adverse outcomes had significantly lower mean rScO_2_ (67 ± 9%) compared with those without adverse outcomes (72 ± 7%), and that rScO_2_ below 50% could be identified as a cut-off point for identifying infants with adverse outcome with an area under the curve of 0.76. Verhagen et al. (39), using pediatric sensors, demonstrated that preterm infants with IVH, compared to those without IVH, had lower median rScO_2_ during the first two weeks following birth, suggesting that lower cerebral blood flow in those with injuries remains present for a longer period than just the first few hours after birth. However, it remains unknown whether this lower blood flow and oxygenation contributed to injury or rather reflects the presence of hemorrhage. In our cohort few infants had rScO_2_ below 50%, therefore we examined the time spent with rScO_2_ below 60% during 72 postdelivery hours. Using this cut-off point in unadjusted analysis we found a positive association between rScO_2TIME<60%_ and brain injury, however after adjusting for weight and GA the significance was lost, but the trend towards positive association remained.

A short-term ARV represents measurement-to-measurement, within-subject variability in the parameter (in our study parameter of interest was rScO_2_) that accounts for the order in which measurements has occurred (31). In cardiovascular research short-term ARV was shown to be an independent risk factor for severity of organ damage (40, 41), cardiovascular morbidity and mortality (30, 42). The precision of estimates from ARV is dependent on frequency of sequential readings (measurements), and in the current study data were recorded every 5–15 s, and ARV was aggregated over 5-minute intervals during 72-hours after delivery. Therefore, our frequency of measurements provides a reliable assessment of rScO_2ARV_ features in regard to the outcome sought. It is well known that inadequate or fluctuating cerebral perfusion and oxygenation contribute to IVH and WMI (10, 12, 38, 43–47). Preterm infants are at high risk for early hemodynamic instability and many factors may contribute to fluctuations in systemic blood pressure in the first few days of life. Moreover, cerebral autoregulation has limited capacity and is thought to be particularly fragile in the immature brain (48). A number of factors that influence vascular reactivity are likely to promote the pressure passive state (hypoxia, hypocarbia, hypercarbia), significantly perturb cerebral blood flow and increase the risk for WMI and intracranial hemorrhage (49–52). The proportion of infants with impaired cerebral autoregulation and increased periods of pressure-passive cerebral circulation appear to be substantial (53, 54). As the pressure-passive state can fluctuate over time and can occur without markedly low blood pressures, it could be readily overlooked with routine monitoring (54). On the other side, increases in systemic blood pressure, especially abrupt increases, could lead to cerebral hyperperfusion and hemorrhagic complications. Since oscillations of systemic blood pressure create variations in regional blood flow which can be assessed with NIRS (55), rScO_2_ ARV emerges as an attractive approach to assess the adequacy of regional brain perfusion and oxygenation. To the best of our knowledge rScO_2ARV_ using NIRS has never been examined in assessing the association between rScO_2_ and brain injury. Our study suggests that increased short-term rScO_2ARV_, early following delivery of preterm infants may be either a predictor or a marker for increased likelihood of brain injury.

### 4.1 Strengths and limitations

A strength of this study is prospective enrollment of consecutive neonates who fulfilled the research criteria. To examine more precisely the relationship between rScO_2_ and brain injury only neonates with a normal cUS on admission were included. Another strength of our study is the use of MRI over cUS to detect the severity and extension of brain injury. Compared with cUS, MRI is more sensitive for detection of low grade IVH, non-cystic WMI, especially punctate white matter lesions which correspond to small periventricular necroses of apparent ischemic or hemorrhagic nature, as well as cerebellar hemorrhage, particularly small punctate hemorrhages (56, 57).

Our study must be interpreted in the context of several limitations. First, we focused on rScO_2_ monitoring in the first 3 days following birth. While most brain injuries (70%) are expected to occur within the first 72 h following birth (13), this monitoring window may not be adequate to capture injuries that occur later (of note, it is estimated that 95% of brain injuries occur by day 7, with a very small additional percentage between days 7 and 10) (24). Our study assumes that the majority of injuries occurred during 72-hour time frame after birth, as well that these injuries may be associated with altered rScO_2_. Second, our study assumes that early occurring brain injuries remain detectable with MRI at TEA, and ignores the possibility that some may have resolved in interim. Third, there was a substantial variability in measured rScO_2_ values which limits the statistical power of consistently detecting differences. Although we found evidence that rScO_2_ in early postdelivery period is associated with brain injury at TEA, as well that the time spent below 60% saturation is associated with increased risk for brain injury, our study is not large enough to provide the exact cut-off point for critical rScO_2_ levels. Therefore, future studies are needed to define critical rScO_2_ values and examine whether interventions designed to optimize rScO_2_ can prevent brain injury in infants.

## 5 Conclusion

In conclusion, our results suggest that features of increased rScO_2_ variability in preterm infants, as well as lower rScO_2MEAN_ and increased percentage of time spent <60% within the first three days following delivery may be associated with increased likelihood for brain injury at TEA. Our study design does not allow to discern whether the observed association between rScO_2_ and brain injury is causative or is rather a marker of its presence. Therefore, our study opens an intriguing question and provides direction for future research.

## Data Availability

The raw data supporting the conclusions of this article will be made available by the authors, without undue reservation.
