# Supplementary figures and images for: Regional cerebral oxygen saturation variability and brain injury in preterm infants
Source: Front Pediatr. 2024 Jul 22;12:1426874. doi: 10.3389/fped.2024.1426874 (PMC11298368; doi:10.3389/fped.2024.1426874)

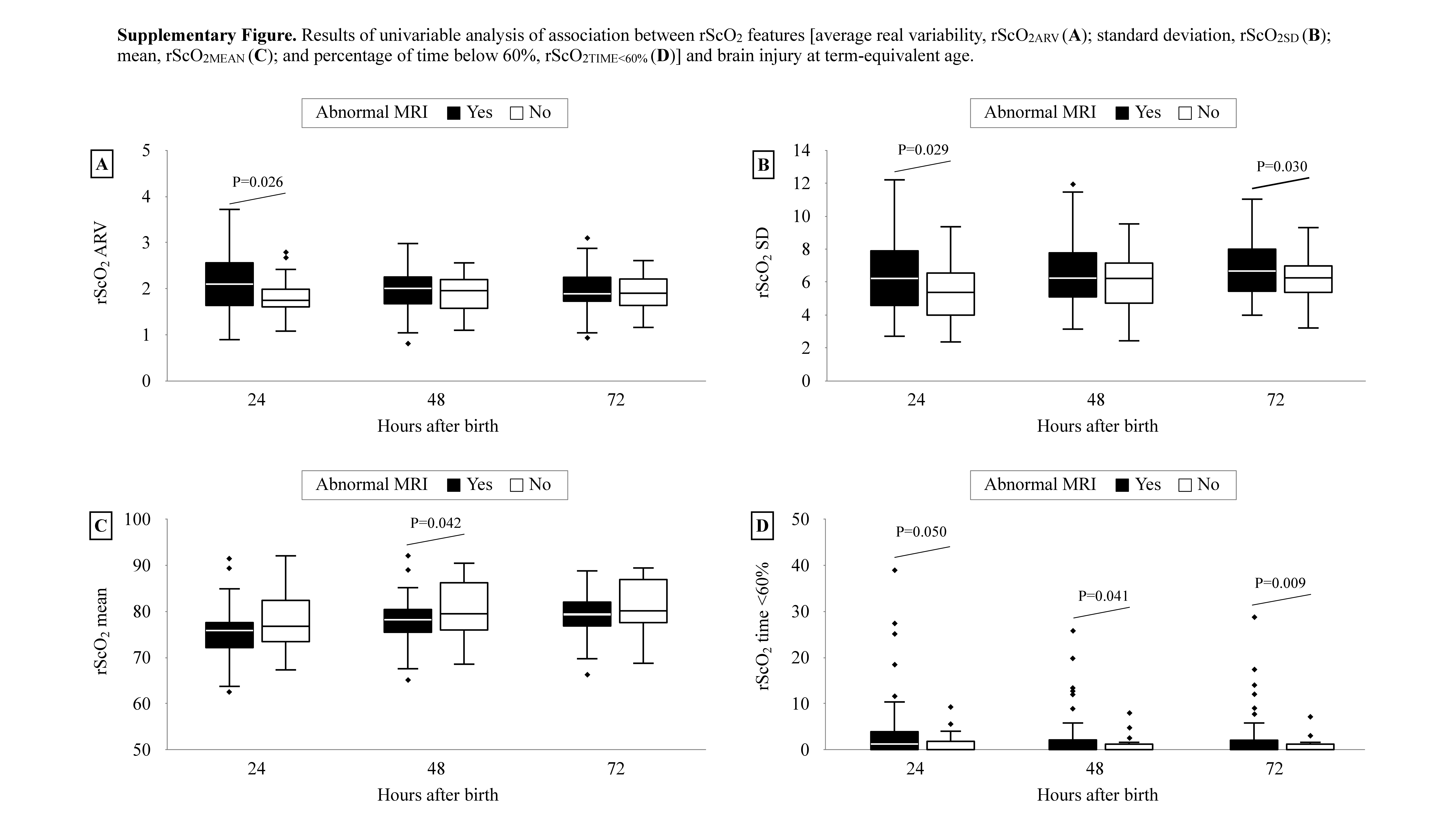

Supplement: Supplementary file 1 [file Image1.tiff]
